# Altered Membrane Structure and Surface Potential in Homozygous Hemoglobin C Erythrocytes

**DOI:** 10.1371/journal.pone.0005828

**Published:** 2009-06-08

**Authors:** Fuyuki Tokumasu, Glenn A. Nardone, Graciela R. Ostera, Rick M. Fairhurst, Steven D. Beaudry, Eri Hayakawa, James A. Dvorak

**Affiliations:** 1 Biophysical and Biochemical Parasitology Section, Laboratory of Malaria and Vector Research, National Institute of Allergy and Infectious Diseases, National Institutes of Health, Bethesda, Maryland, United States of America; 2 Research Technology Branch, National Institute of Allergy and Infectious Diseases, National Institutes of Health, Bethesda, Maryland, United States of America; 3 Malaria Pathogenesis and Human Immunity Unit, Laboratory of Malaria and Vector Research, National Institute of Allergy and Infectious Diseases, National Institutes of Health, Bethesda, Maryland, United States of America; 4 International Research and Educational Institute for Integrated Medical Sciences (IREIIMS), Tokyo Women's Medical University, Tokyo, Japan; Federal University of São Paulo, Brazil

## Abstract

**Background:**

Hemoglobin C differs from normal hemoglobin A by a glutamate-to-lysine substitution at position 6 of beta globin and is oxidatively unstable. Compared to homozygous AA erythrocytes, homozygous CC erythrocytes contain higher levels of membrane-associated hemichromes and more extensively clustered band 3 proteins. These findings suggest that CC erythrocytes have a different membrane matrix than AA erythrocytes.

**Methodology and Findings:**

We found that AA and CC erythrocytes differ in their membrane lipid composition, and that a subset of CC erythrocytes expresses increased levels of externalized phosphatidylserine. Detergent membrane analyses for raft marker proteins indicated that CC erythrocyte membranes are more resistant to detergent solubilization. These data suggest that membrane raft organization is modified in CC erythrocytes. In addition, the average zeta potential (a measure of surface electrochemical potential) of CC erythrocytes was ≈2 mV lower than that of AA erythrocytes, indicating that substantial rearrangements occur in the membrane matrix of CC erythrocytes. We were able to recapitulate this low zeta potential phenotype in AA erythrocytes by treating them with NaNO_2_ to oxidize hemoglobin A molecules and increase levels of membrane-associated hemichromes.

**Conclusion:**

Our data support the possibility that increased hemichrome deposition and altered lipid composition induce molecular rearrangements in CC erythrocyte membranes, resulting in a unique membrane structure.

## Introduction

Unstable hemoglobin (Hb) variants, such as HbC, sickle HbS, and unpaired beta globin chains present in α-thalassemic states, impart a greatly increased level of oxidative stress on erythrocytes that enhances the oxidative denaturation of Hb [1]–[5]. Excess reactive oxygen species and free radicals oxidize Hb to metHb and then further to hemichrome, a low-spin ferric hemoglobin derivative that binds to and clusters erythrocyte membrane protein band 3 by a process associated with erythrocyte senescence [6]–[8]. HbC associates with erythrocyte membranes at a 5-fold greater rate than normal HbA [9] and binds more tightly to the inner leaflet, where it is believed to cause more extensive clustering of band 3 [10]. These changes in membrane structure, as well as dehydration-induced HbC crystallization and increased internal viscosity, are believed to play some role in the mild anemia that homozygous CC individuals experience as a result of accelerated erythrocyte turnover [11]–[13].

Membrane-bound hemichromes are thought to serve as sources of additional oxidative damage through iron-catalyzed production of hydroxyl radical (OH^·^) and the liberation of heme and free iron [14]–[18]. Indeed, free non-heme iron has been shown to accumulate in HbS and thalassemic erythrocyte membranes [19], [20]. These processes are believed to enhance membrane lipid peroxidation and protein cross-linking [21], and phosphatidylserine (PS) externalization [22]. Thus, hemichromes and hemichrome-induced processes may modify the architecture of erythrocyte membranes. Since these processes occur at greater levels in homozygous CC erythrocytes, they have the potential to produce marked changes in the two-dimensional membrane matrix of these cells, which could alter their membrane fluidity and impede the lateral diffusion or mobility of their membrane components [23]. Compared to AA erythrocytes, we hypothesized that CC erythrocytes have marked differences in their membrane lipid profile, two-dimensional membrane matrix, and macroscopic biophysical and electrochemical properties. To test these hypotheses, we compared the membrane lipid and raft [24] composition of erythrocytes obtained from AA and CC individuals using HPLC-based analyses of extracted erythrocyte lipids and immunoblot analyses of detergent-solubilized membrane fractions. We also employed an electrophoretic mobility assay to measure the net membrane potential, known as zeta potential (ZP), of individual AA and CC erythrocytes to determine whether any differences in their membrane matrices might be associated with alterations in their whole-cell physiology.

Rafts (or membrane microdomains) are putative membrane entities that are proposed to have important physiological functions [25], such as signal transduction [26], and their molecular composition can be determined by analyzing detergent-resistant membrane (DRM) fractions. While the functions of rafts in erythrocytes have not been definitively elucidated, some raft-associated GPI-anchored proteins have been implicated in immune-mediated clearance of erythrocytes [27]. While the structure of rafts and their contribution to the physical properties of live cell membranes continue to be clarified, analyses of DRM fractions are useful in comparing AA and CC erythrocyte membranes for differences in lipid packing conditions and lateral protein distributions.

Significant modifications of rafts, together with membrane-associated hemichromes and plasma protein aggregates [28], would be predicted to change the whole-cell net charge of CC erythrocytes. This can be determined by comparing ZP measurements of AA and CC erythrocytes. The ZP of a cell is a measure of the electrochemical potential of its membrane, as determined by the amount and sign of associated ions. Among numerous charge-bearing molecules in the erythrocyte membrane, sialic acid contributes substantially to the high net negative charge on the surface of erythrocyte membranes, and removal of sialic acid by neuraminidase treatment results in erythrocyte aggregation [29]. Sufficiently high negative charge (high ZP) of the erythrocyte surface is believed to suppress cellular aggregation [30] and enable erythrocyte populations to maintain stable suspensions. ZP is thus a useful parameter to study the impact of membrane modifications on the net charge and adhesive properties of cell surfaces.

Employing these chemical and biophysical methods, we found that CC erythrocytes show characteristic alterations in both nanoscopic and macroscopic membrane properties that may affect intrinsic protein distributions and/or functions as well as intercellular interactions.

## Materials and Methods

### Erythrocytes

Erythrocyte acquisition, preparation and hemoglobin phenotype determination were described previously [31]. Blood sampling was conducted under a protocol approved by the NIAID Institutional Review Board.

### Lipid extraction

Extraction of lipids was performed according to published methods [32]. Samples were prepared under Argon gas to avoid lipid oxidation during the extraction procedure. 600 µL of a 50% erythrocyte suspension was slowly mixed drop-wise with 4.5 mL of methanol while gently vortexing in glass centrifuge tubes. After 30 min, 3 mL of chloroform were added drop-wise with gentle vortexing. The suspension was centrifuged at ≈1000×*g* for 5 min and the supernatant was passed through a 0.2 µm PTFE syringe filter (Nalge Nunc, Rochester, NY). Stigmasterol and 1,2-dipalmitoleoyl-*sn*-glycero-3-phosphocholine (di16∶1 PC) were added as internal standards [33]. Extracts were stored under Argon gas in glass centrifuge tubes at −20°C. Solvent was removed in a nitrogen evaporator and the residue was dissolved in 120 µL of hexane:isopropanol:water (6∶8∶1). Samples were centrifuged at 10,000 rpm for 10 min to remove particulates.

### Purification and quantification of lipids

Lipid classes were purified and quantified using modifications of established methods [33]. The crude sample was injected into a 4×250 mm Lichrospher Si 100 5-micron column (Merck, Darmstadt, Germany) on an Agilent 1100 series analytical HPLC (Agilent, Santa Clara, CA) and developed isocratically with hexane:isopropanol∶25 mM potassium phosphate (pH 7):ethanol:acetic acid (369∶475∶56∶100∶0.1). Effluent was monitored at 205 nm. Fractionated lipids were first pooled by class. The internal standard dimyristoyl phosphatidylcholine (PC), (Avanti Polar Lipids, Alabaster, AL) was then added to the phosphatidyl-ethanolamine (PE), phosphatidylserine (PS) and phosphatidylinositol (PI) pools and the internal standard N-lignoceryl ceramide (Avanti Polar Lipids) was added to the sphingomyelin (SM) pool. Solvent was removed in a model N-Evap111 nitrogen evaporator (Organomation Associates, Berlin, MA). The samples were desalted and hydrolyzed with phospholipase C or sphingomyelinase (Sigma-Aldrich, St Louis, MO). The diacylglycerols and cholesterol fractions were benzoylated, extracted, and dried under N_2_ gas. Samples were then dissolved in 100 µL of methanol:chloroform (10∶2) and centrifuged at 10,000 rpm to remove particulates. Typically, 3 to 6 µL of sample was analyzed by injection into a Zorbax 0.5×150 mm SB-C18 5 micron column (Agilent) developed isocratically with methanol:water:acetonitrile (94.25∶5∶1.75) at a flow rate of 10 µL/min on an Agilent 1100 series analytical HPLC (Agilent). Effluent was monitored at 230 nm. During this step, peak areas were determined and the internal standard was separated from the extracted lipids.

We estimated the amount of benzoylated lipids present using the following equation:
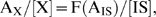
(1)where A_x_ is the integrated peak areas of the unknown membrane-derived diacylglycerols, ceramides, or sterols at 230 nm, [X] is the mole quantity of lipid analytes, F is the detector response ratio between analyte and internal standard at 230 nm, A_IS_ is the integrated peak area of the internal standard at 230 nm, and [IS] is the mole quantity of internal standard added to the sample. F was taken as 1 for these analyses since both the analytes and internal standards were labeled by identical chemistry with benzoyl groups. Differences in benzoyl absorptivities between analytes and standards were assumed to be absent due to nearly identical structures and chemical groups between the two samples.

### Flow cytometry

Washed erythrocytes (1×10^6^/mL) were reacted in HEPES buffer with fluorescein isothiocyanate (FITC)-conjugated annexin-V (Invitrogen, Carlsbad, CA) for 20 min at RT, followed by washing with HEPES buffer. FITC signal was detected using 488 nm excitation light on a FACS Calibur instrument (BD, Franklin Lakes, NJ). For each experiment, fluorescence signals of unstained erythrocytes for each hemoglobin type (autofluorescence) were measured and used to adjust the fluorescence intensity of Annexin-V stained erythrocytes. Data were analyzed using FlowJo software version 7.2.5.

### Detergent-resistant membrane analysis

DRM samples were prepared by an established method [34], [35]. 200 µL of packed erythrocytes were lysed with 800 µL of ice-cold 1% Triton X-100 in TBS, containing an antiproteinase cocktail (Complete®; Roche Diagnostics, Mannheim, Germany). Erythrocytes were lysed completely by pipetting several times and incubated on ice for 20 min. The lysate was mixed with an equal volume of ice-cold 0.2 M Na_2_CO_3_ [34], 80% sucrose in TBS. The mixture (2 mL) was transferred to the bottom of a Beckman 14×89 mm polyallomer ultracentrifuge tube (Beckman Coulter, Fullerton, CA), overlaid by 6 mL of 30% sucrose in TBS, and followed by overlaying 3 mL of 10% sucrose in TBS. The gradient was centrifuged with a SW41-Ti rotor (Beckman Coulter) at 39,000 rpm for 18 h at 4°C. Fractions were collected from the top of the tube in 300 µL aliquots and stored at -20°C until used.

### SDS gel electrophoresis

Protein distributions over a total of 36 sucrose gradient fractions were analyzed using 4–12% Bis-Tris acrylamide gel (Invitrogen) under reducing conditions. For each protein analysis, each lane was loaded with the same volume of AA and CC sample (10–20 µL). Proteins were transferred to a PVDF membrane and blocked overnight in blocking buffer (Sigma-Aldrich). Proteins were probed by incubating the membrane with 0.1–0.2 µg/mL of monoclonal antibody (diluted in blocking buffer) specific for each protein. The membrane was washed 3 times with PBS/0.1% Tween-20 for 6 min, reacted with secondary antibody in the blocking buffer for 1 h, washed 3 times with PBS/0.1% Tween-20 for 6 min, and developed by Super Signal West Pico chemiluminescence solution (Invitrogen).

### Dot blot analysis

Relative amounts of band 3 and CD47 in each sucrose fraction and their sample variations were determined by dot blot analysis. An equal volume (0.8 µL) of each fraction was directly adsorbed onto a hydrated nitrocellulose membrane (0.45-mm pore size) (Invitrogen). The membrane was blocked and reacted with monoclonal antibody, secondary antibody, and chemiluminescence solution using the same protocol described for SDS gel electrophoresis. The dot patterns were optically scanned and saved as 8-bit gray scale TIFF file. Density data were collected by pixel intensity analysis using Image Pro version 6.3 (Mediacybernetics, Bethesda, MD).

### Induction of hemichromes in AA erythrocytes

Formation of hemichromes and other hemoglobin degradation products was induced by treating 600 µL of a 50% AA erythrocyte suspension with 1 mM sodium nitrite (NaNO_2_) for 1 h at 37°C in HEPES buffer. Erythrocytes were then washed twice with HEPES buffer and membrane ghosts were prepared by lysing erythrocytes at 4°C in Tris-HCl/EDTA pH 8.0 containing Complete® protease inhibitor (Roche Diagnostics). Membrane pellets were extracted with detergent as described previously [36]. Hemichrome levels were quantified using absorption at 560, 577, and 630 nm [37] and expressed as nmol/mL membrane.

### Zeta potential measurements

Zeta potential (ZP) analysis was performed with a Zeecom ZP analyzer (Microtec, Japan). Erythrocytes in RPMI were washed and suspended to 10% in HEPES-buffered saline, pH 7.05. ZP was measured by electrophoresis of erythrocytes through the glass chamber with applied voltage of 20–30 V. The mobility of erythrocytes detected by a microscopic tracking system was used for calculating ZP by applying the Smoluchowski formula [29], 
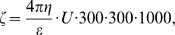
(2)where 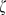 is ZP, 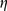 is viscosity of solution, 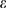 is dielectric constant, and *U* is electrophoretic mobility. 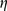 and 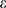 are approximated as those of water: a deviation of the dielectric constant of HEPES buffer was negligible (≈2%) [38]. Two constants in the equation were required to convert CGS units used in the equipment to MKS units. Statistical analyses of the data were performed with Origin 7.5 (OriginLab, Northampton, MA).

## Results

### Lipid profile analysis

Erythrocyte membranes contain most of the major lipids that are present in other biological membranes, including phosphatidylcholine (PC), sphingomyelin (SM), phosphatidylethanolamine (PE), phosphatidylserine (PS), phosphatidyl-inositol (PI), and cholesterol. These lipids are distributed asymmetrically in the membrane, with PC and SM found mainly in the outer leaflet and PE and PS found mainly in the inner leaflet. The relative amounts of these lipids and their asymmetric distributions are important in maintaining the integrity of the erythrocyte membrane and cytoskeleton.

**Figure 1:**
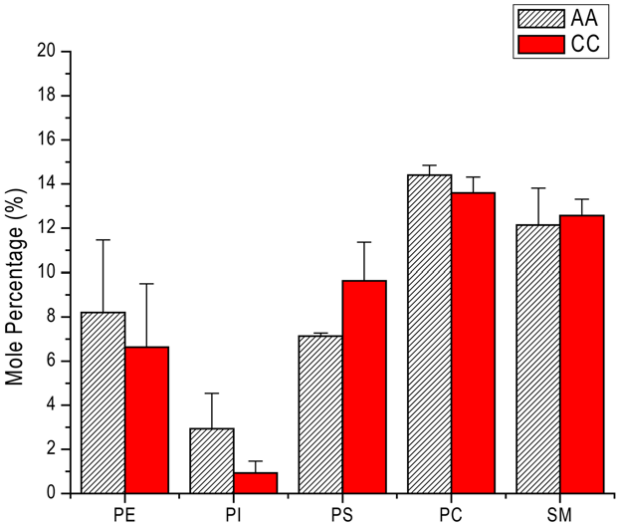
Lipid composition of AA and CC erythrocytes. All mean values are shown as mole % of total lipids including cholesterol. Error bars represent SEM (n = 3).

To begin comparing the biochemical and biophysical properties of AA and CC erythrocytes, we first studied the lipid composition of their membranes. Of the many published protocols for erythrocyte lipid extraction, we chose the method of Wang and Gustafson [32] since it produces stable extraction efficiencies for each lipid class. Extracted lipids were separated by HPLC and analyzed by a semi-quantitative method based on internal standards introduced in the sample. Figure 1 compares the mean (±SEM, n = 3) mole percentages of each lipid class in AA and CC erythrocytes. We found no significant difference in the amounts of PC, SM, PE, and PI extracted from AA and CC erythrocytes (Fig. 1). However, the data suggested that the amount of PS extracted from CC erythrocytes was greater than that extracted from AA erythrocytes. One possible explanation for this finding is that increased levels of PS flip to the outer leaflet of the CC erythrocyte membrane, where it might be more efficiently extracted.

**Figure 2:**
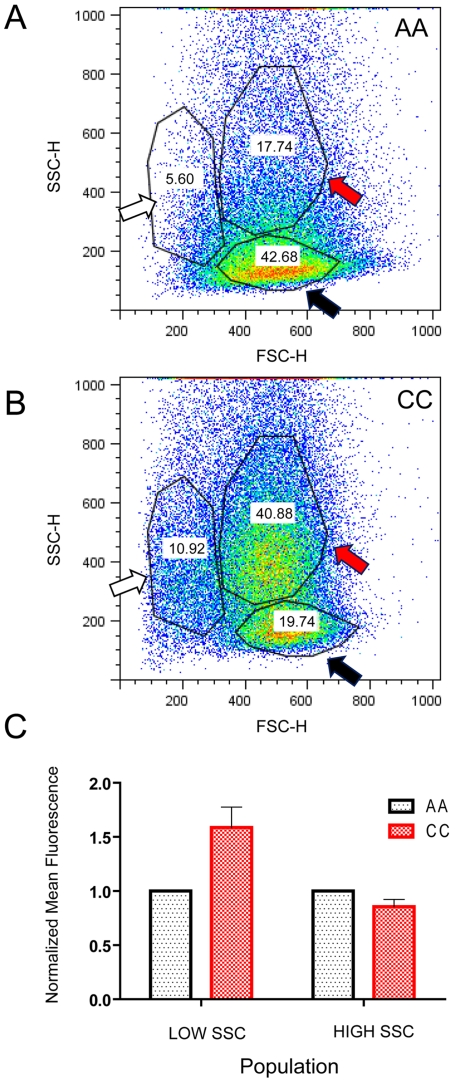
Flow cytometry analyses of phosphatidylserine on the surface of unfixed AA and CC erythrocytes. Erythrocytes were reacted with FITC-labeled Annexin-V. Subpopulations of AA (A) and CC (B) erythrocytes were gated and the percentage of total erythrocytes within each gate was calculated. Normalized mean fluorescence intensities of the low and high side scatter populations (black and red arrows, respectively) are shown in panel C. Error bars represent SEM (n = 4).

PS externalization in human erythrocyte membranes is associated with erythrocyte senescence [39], [40]. In this process, oxidative stress increases cytosolic Ca^2+^ concentration [41], [42] and activates Ca^2+^-sensitive scramblase [43], [44], which breaks up PS asymmetry. Externalized PS and associated bridging molecules are specifically recognized by macrophages that phagocytose the senescent erythrocyte [45]–[47]. Whether increased PS externalization in CC erythrocytes contributes to the accelerated removal of these cells *in vivo* has not been investigated. To explore this hypothesis, we used FITC-labeled annexin-V in a flow cytometry assay to compare the amounts of externalized PS on the surface of unfixed AA and CC erythrocytes. We first examined the forward scatter (FSC) and side scatter (SSC) profiles of AA and CC erythrocytes (Figs. 2A and 2B). While FSC generally represents differences in cell refractive index and is proportional to cell size, SSC reflects cell granularity and surface irregularity. We identified three subpopulations of CC erythrocytes that differed significantly in their FSC or SSC profiles. The proportion of CC erythrocytes showing high SSC and normal FSC (≈400–600) was increased compared to AA erythrocytes (red arrows, ≈41% *vs.* ≈18%). The proportion of CC erythrocytes showing high SSC and low FSC (≈100–300) was also increased compared to AA erythrocytes (white arrows, ≈11% *vs.* ≈6%). The increased proportions of CC erythrocytes with high SSC values may reflect known morphological and membrane structural abnormalities in these cells, such as poikilocytosis and hemichrome deposition [9].

Differences in scatter profiles may indicate significant alterations in cell morphology, surface area, and internal physical properties. To investigate how these differences might influence the levels of externalized PS, we compared the mean fluorescence intensity (MFI) of annexin V-FITC labeled cells in two subpopulations (Figs. 2A and 2B, black and red arrows) with similar FSC profiles but dramatically different SSC profiles. In the low SSC population, the MFI for CC erythrocytes was 1.6±0.2 (mean±SEM, n = 4, *P* = 0.05, one sample *t* test of the mean) fold higher than AA erythrocytes (Fig. 2C), indicating higher levels of externalized PS. In contrast, the MFI for CC erythrocytes in the high SSC population was slightly lower that AA erythrocytes, but this was not statistically significant (0.86±0.07, n = 4, *P* = 0.12) (Fig. 2C). No difference was observed in the MFIs (not shown) of AA and CC erythrocytes in the low FSC population (Figs. 2A and 2B, white arrows). These data suggest that the normal asymmetric distribution of PS is perturbed in a substantial proportion of CC erythrocytes, a phenomenon that could affect the lateral distribution of other lipids.

### Detergent resistant membrane (DRM) analyses

**Figure 3:**
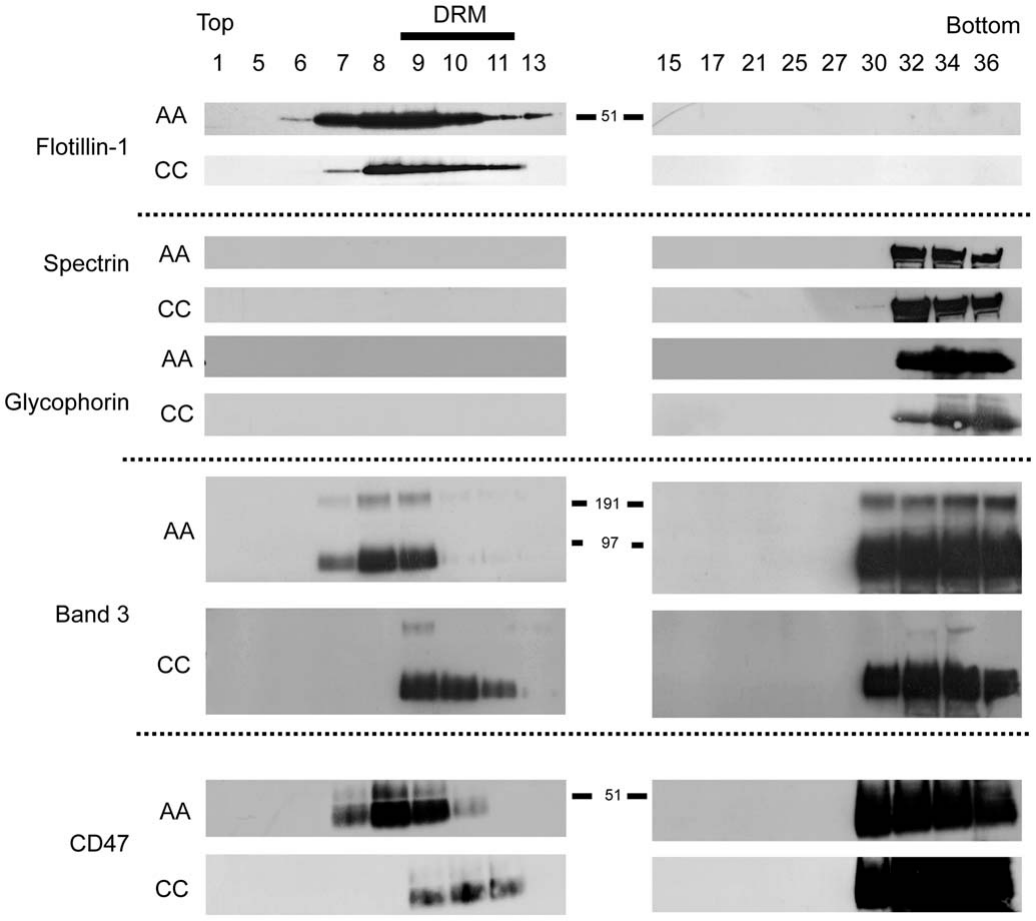
Detergent-resistant membrane analyses of AA and CC erythrocytes. Proteins from 18 fractions (out of a total of 36 fractions) were separated by SDS-PAGE under denaturing conditions, transferred to PVDF membrane, and probed with protein-specific monoclonal antibodies.

Changes in lipid distribution are believed to alter membrane phase behaviors and nanoscopic membrane domains. To determine whether AA and CC erythrocytes differ in the lateral distribution of membrane components, we studied the composition of their detergent-resistant membranes (DRM). After detergent treatment, erythrocyte membranes were fractionated on sucrose gradients. DRMs usually appear as a white cloudy band concentrated at about 30% from the top of an 11-mL volume gradient [48], [49]. Erythrocyte DRM fractions contain flotillin-1, flotillin-2, ganglioside GM1, stomatin, and some band 3 molecules; cytoskeletal proteins generally do not appear in DRM [34], [49], [50]. We found that the macroscopic appearance (not shown) and position of AA and CC erythrocyte DRM bands in fractions 9–11 (indicated by bar in Fig. 3) were indistinguishable in sucrose gradients. To determine whether the membrane microdomains of CC erythrocytes were modified, we first isolated 300-µL fractions from the 11-mL sucrose gradient (36 fractions). We then used immunoblot analysis to detect differences in the distribution of the DRM marker protein flotillin-1 over the various fractions. We detected flotillin-1 in fractions 6–13 from AA erythrocytes but only in fractions 7–11 from CC erythrocytes, indicating that flotillin-1 is more concentrated in the DRMs of CC erythrocytes (Fig. 3). As shown previously for AA erythrocytes [34], [35], spectrin and glycophorin were found exclusively in the bottom fractions of CC erythrocyte preparations (Fig. 3).

Similar to previous studies of AA erythrocytes [49], [50], we detected most band 3 proteins in the bottom fractions 30–36 and a smaller amount of band 3 in the DRM fractions 7–9 (Fig. 3). In four independent immunoblot analyses, we detected band 3 exclusively in DRM fractions 7–9 from AA erythrocytes. In both DRM and bottom fractions, band 3 was present as ≈95 kDa monomers and ≈191 kDa dimers, despite the use of reducing conditions in the processing of samples. Although no structural differences have been described to explain the persistence of band 3 dimers in samples processed under reducing conditions, this may be due to the strong ‘interlocking’ structure of band 3 dimers [51]. In CC erythrocyte samples, we also detected most band 3 proteins in the bottom fractions 30–36. In four independent immunoblot analyses, however, we found the remainder of band 3 to be exclusively in DRM fractions 9–11 (Fig. 3). These data indicate that band 3-containing DRM are denser in CC erythrocytes than those of AA erythrocytes. Extensive band 3 oligomerization in CC erythrocyte membranes [10] may help explain this finding. It may also explain why the relative proportions of band 3 dimers in both DRM and bottom fractions seem to be lower in CC than in AA erythrocytes (Fig. 3). Specifically, extensive band 3 oligomerization may reduce the amount of band 3 dimers that can be successfully extracted from CC erythrocytes and analyzed [10].

In the erythrocyte membrane, band 3 interacts with the Rh-complex [52]. CD47, a component of the Rh-complex, serves as an immunological ‘self’ marker that prevents the premature phagocytic removal of erythrocytes from the bloodstream [53], [54]. To explore the possibility that band 3 modifications in CC erythrocytes cause secondary structural alterations in the Rh-complex, we compared the lateral distribution of CD47 in sucrose gradient fractions from AA and CC erythrocytes. In four independent experiments, we found that the distribution patterns of CD47 were nearly identical to those of band 3 in AA and CC erythrocytes (Fig. 3). These data suggest that band 3 and CD47 reside in the same membrane microdomain in both AA and CC erythrocytes, and that the packing of band 3 and Rh-complexes may be tighter in the DRM of CC erythrocytes.

**Figure 4:**
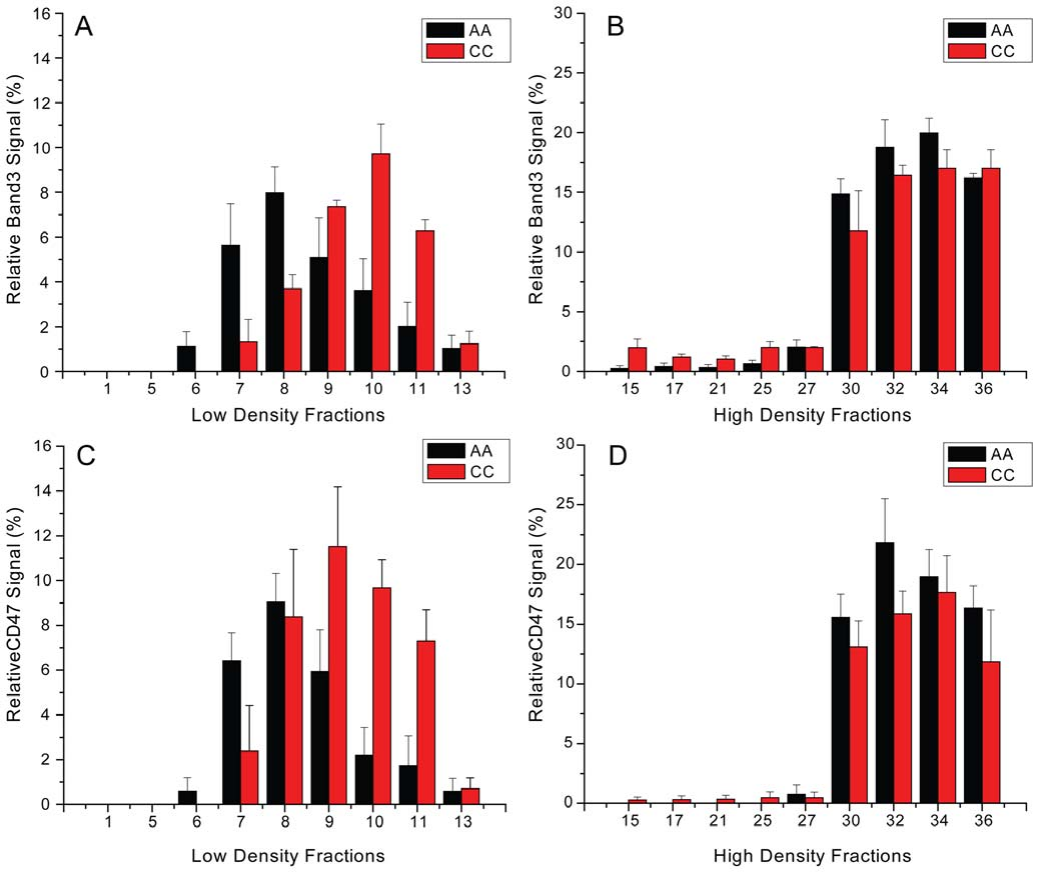
Distributions of relative band 3 and CD47 signals from dot blot analyses. For each protein, the sum of signals from all fractions was set as 100%. Band 3 (A, B) and CD47 (C, D) signals showed similar distributions in the high density fractions of AA and CC samples. However, differences in band 3 and CD47 signal distributions were observed in the low density fractions of AA and CC samples. Higher density signals for both proteins in CC samples appeared in fractions 9–11, which correspond to visible DRM.

To quantitatively analyze the protein distribution patterns for band 3 and CD47, we performed dot blot analyses. In four independent experiments, we found differences in the signal distribution pattern in the low density fractions of AA and CC erythrocyte samples (Figs. 4A and 4C). Band 3 signal peaked in fraction 8 from AA samples and in fraction 10 from CC samples (Fig. 4A), and CD47 signal peaked in fraction 8 from AA samples and in fraction 9 from CC samples (Fig. 4C). In contrast, signal distributions for both band 3 and CD47 in the bottom fractions were essentially indistinguishable (Figs. 4B and 4D). These data provide additional evidence that band 3/CD47-containing DRM are denser in CC erythrocytes than those of AA erythrocytes.

**Figure 5:**
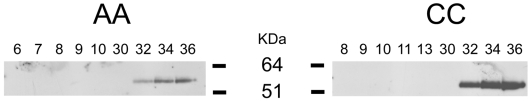
Analysis of membrane-associated IgG in AA and CC erythrocytes. Proteins from 9 fractions (out of a total of 36 fractions) were separated by SDS-PAGE under denaturing conditions, transferred to PVDF membrane, and probed with a monoclonal antibody specific for human IgG.

Erythrocyte senescence is believed to induce formation of a band 3 ‘neoantigen,’ to which autologous IgG binds and opsonizes the cell for phagocytic removal from the bloodstream by macrophages [6], [8], [55], [56]. While CC erythrocytes contain significantly more membrane-associated IgG than AA erythrocytes [28], [31], nearly all of this IgG co-localizes with other plasma proteins (IgM, IgA, complement, albumin) within large intracellular aggregates and is not found on the cell surface. We considered it possible, however, that small amounts of IgG might modulate membrane microdomains by directly binding externalized regions of band 3 and thereby altering the diffusion and/or distribution of this protein within microdomains. To test this possibility, we tested band 3-containing fractions of AA and CC erythrocytes for the presence of IgG. Interestingly, we found IgG only in the densest membrane fractions from both cell types (Fig. 5), with amounts of IgG much higher in CC than in AA erythrocytes. It is likely that most of these IgG molecules are components of membrane-associated plasma protein aggregates that have been observed in CC erythrocytes [28]. Whether any of these IgG molecules bound membrane microdomains and increased their density is possible, but very unlikely given the lack of flotillin-1 detection in the same fractions.

### Zeta potential of erythrocyte membrane

**Figure 6:**
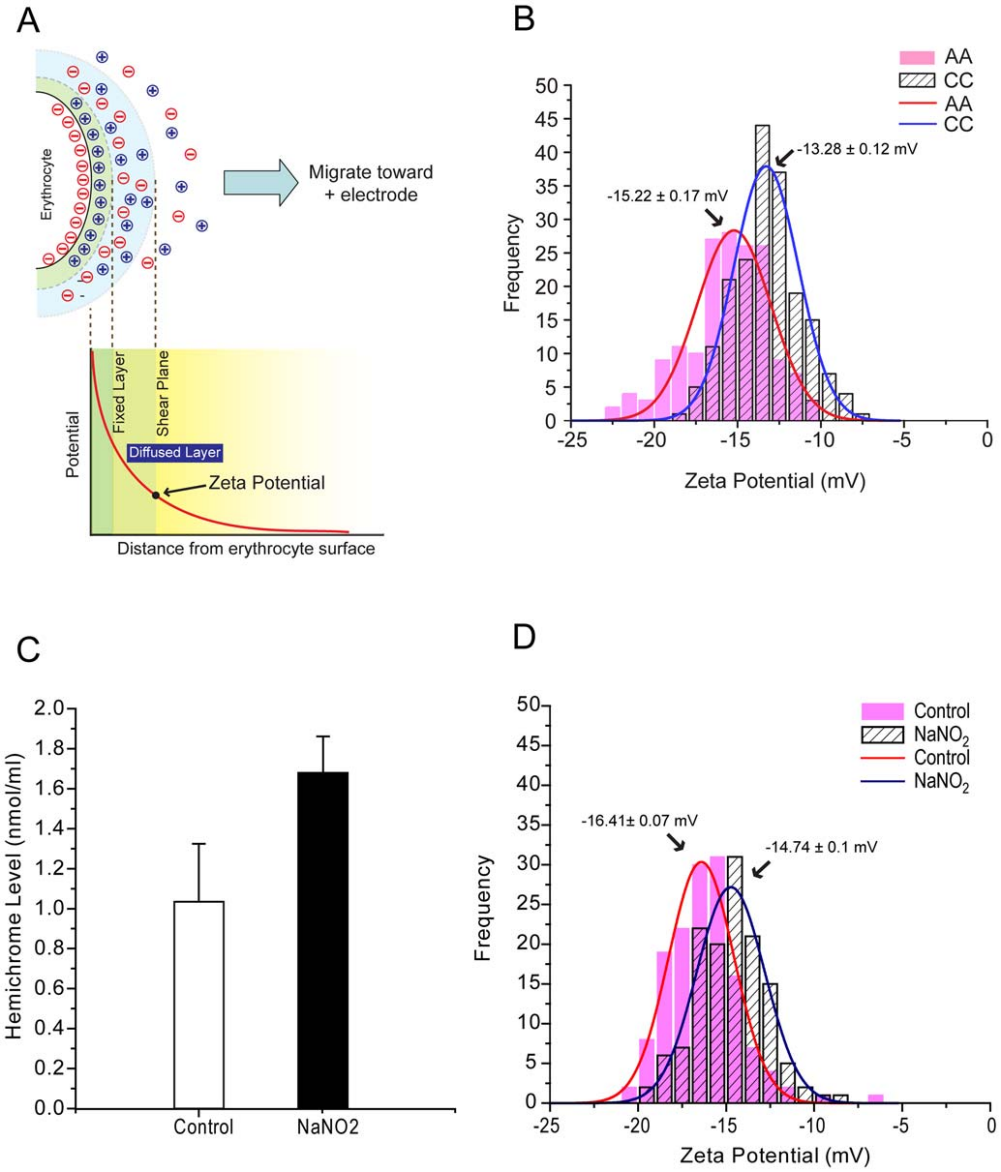
Zeta potential (ZP) analyses. (A) Diagram of ZP principle. ZP is defined as the electrochemical potential at the shear plane. Outside the shear plane, ions are not closely associated with the internal ion cloud. (B) ZP measurements from AA and CC erythrocyte populations. Peak values were estimated by Gaussian fitting the histogram. (C) Levels of membrane-associated hemichromes (mean±SD) in control and NaNO_2_-treated AA erythrocytes. For reference, native CC erythrocytes show 1.8-fold greater hemichrome levels than native AA erythrocytes [31]. (D) ZP measurements from control and NaNO_2_-treated AA erythrocyte populations.

The lateral organization of membrane components largely depends on the relative amounts and intermolecular interactions of proteins and lipids, and the attachment of extracellular proteins. These and other factors determine the net membrane potential, which can be measured as zeta potential (ZP). Measurement of ZP is an easy and relatively quick way to detect molecular changes that have occurred on the membrane surface of live cells. The basic principle of ZP is shown in Figure 6A. Higher net surface charge attracts more counter ions in the environment and form fixed ion clouds. In turn, this steady ion cloud attracts ions having the same sign as those present at the cell surface, creating a diffused layer composed of a mixture of cations and anions. Within the diffused layer, Brownian motion of erythrocytes creates a shear plane which separates ions strongly associated with the fixed layer from the rest of population. The potential at the shear plane is defined as ZP.

To evaluate the influence of the membrane modifications we observed in CC erythrocytes on the net cell surface charge, we compared the ZP of individual AA and CC erythrocytes. We measured the electrophoretic mobilities of individual erythrocytes at the applied voltage of 30 V to estimate ZP values (see Materials and Methods for conversion from mobility to ZP). Both AA and CC erythrocytes showed distributions of ZP over a range of 12 mV (Fig. 6B). Statistical analyses using Gaussian fitting showed that the peak ZP (±SEM) of AA erythrocytes was −15.21±0.17 mV (Fig. 6B). In contrast, the peak ZP of CC erythrocytes decreased to −13.28±0.12 mV, approximately 2 mV less negative than AA erythrocytes (n = 165 for AA, n = 189 for CC, *P*<0.001) (Fig. 6B). Although decreases in the negative surface charge of CC erythrocytes suggested they might have an increased tendency to aggregate due to reductions in repulsive Coulomb forces, we did not observe CC erythrocyte aggregation in this ZP range.

Among the many modifications observed in CC erythrocytes, the presence of membrane-bound hemichromes is likely to alter molecular interactions within the membrane. To determine whether increased levels of membrane-bound hemichromes might have affected the ZP of CC erythrocytes, we induced hemichromes and other hemoglobin degradation products in AA erythrocytes with sodium nitrite (NaNO_2_). At certain concentrations, NaNO_2_ promotes not only the peroxidation of lipids [57] but also the conversion of oxyhemoglobin (Fe^2+^) to methemoglobin (Fe^3+^) [58], [59], which spontaneously converts to hemichromes at a rate that depends on its own tertiary structure [60]. Treatment of AA erythrocytes with NaNO_2_ increased hemichromes by 65% (1.03±0.29 for control AA *vs.* 1.68±0.18 for NaNO_2_-treated AA, n = 6, *P* = 0.0009) (Fig. 6C) to a level previously reported for native CC erythrocytes (Fig. 6C legend) [31]. Figure 6D shows that the ZP of NaNO_2_-treated AA erythrocytes is 1.6 mV less negative than that of untreated AA erythrocytes (n = 143 for AA, n = 133 for CC, *P*<0.001). The degree and direction of ZP modifications in NaNO_2_-treated AA erythrocytes were similar to those observed in native CC erythrocytes (Fig. 6B). Therefore, oxidation-induced hemichromes, which bind the inner leaflet of the erythrocyte membrane and cluster band 3, may in part account for the observed reduction in ZP and membrane microdomain modifications in CC compared to AA erythrocytes.

## Discussion

### Increased levels of externalized PS in a subset of CC erythrocytes

We found that the amounts of most membrane lipids extracted from AA and CC erythrocytes were similar, suggesting that our extraction procedure was able to release lipids from both the inner and outer leaflet. By contrast, the amount of PS we extracted from CC erythrocytes was greater than for AA erythrocytes. An increase in PS yield suggested the possibility that some membrane PS was more easily extracted from CC than AA erythrocytes, for example, by flipping to the outer leaflet where it would be free from hemichromes and other hemoglobin degradation products that might resist efficient extraction. Indeed, this interpretation is consistent with our finding of increased annexin-V signals in a subpopulation of CC erythrocytes. This subpopulation showed similar FSC and SSC profiles to that of the main AA subpopulation (Figs. 2A and B, black arrows). These data suggest that this subset of CC erythrocytes has an AA-like physical condition, which may include similar levels of membrane-associated hemichromes. Relatively low levels of HbC hemichromes in this subpopulation may allow more efficient externalization of PS as a result of oxidation and other processes. Increased PS exposure has been reported in a variety of erythrocytes with altered physical conditions, such as senescent erythrocytes [61], [62] and subpopulations of erythrocytes from individuals with homozygous HbS (sickle cell disease) [16], beta-thalassemia [22], or type-1 diabetes [63]. However, comprehensive studies will be required to determine the relationships between the particular physical properties of CC erythrocytes and their levels of annexin-V-accessible, biologically-active PS.

### Significant alterations in membrane microdomains of CC erythrocytes

We found narrower distributions of flotillin-1 in the DRM fractions of CC erythrocyte membranes, indicating that the DRM are less heterogeneous in density and thus may be packed differently than those in AA erythrocytes. Differences in both DRM protein composition and levels of membrane-associated hemichromes can directly affect the structure of lipid domains. We found that DRM-associated band 3 and CD47 are located in higher sucrose density fractions in CC compared to AA erythrocytes, suggesting that these proteins might be more extensively clustered or complexed with other DRM proteins. Excessive hemichrome attachment may also affect domain structure by modulating the diffusion of proteins and lipids in CC erythrocyte membranes. By inducing modifications in the phase behaviors and domain structures of the inner membrane, hemichromes may create large differences in the structures of inner and outer leaflets. The association of plasma protein aggregates with CC erythrocyte membranes could facilitate this effect. Like CC erythrocytes, SS erythrocytes containing elevated levels of sickle HbS hemichromes show distributions of flotillin-1 that concentrate more narrowly in sucrose gradient DRM fractions (unpublished data). This finding, and reports of significant membrane protein redistributions in SS erythrocytes [64], suggest that similar membrane alterations may occur in CC erythrocytes. Direct observations of individual molecules at high speed and resolution (e.g., single particle tracking) will likely be required to investigate how AA, CC, and SS erythrocytes differ in the distribution and diffusion patterns of membrane components.

We observed that band 3 and CD47 distribute to both DRM fractions and the bottom fractions of sucrose density gradients, indicating that each of these membrane proteins has two different forms. It is possible that each protein is present as a relatively mobile population and also as a less mobile population that interacts with the cytoskeleton. Compared to AA erythrocyte membranes, we detected lesser amounts of band 3 dimers in CC erythrocyte membranes. These reductions in band 3 signal were observed in both in DRM and bottom fractions. It is possible that band 3 in CC erythrocyte membranes is more tightly clustered and thus relatively more resistant to detergent extraction. The distribution patterns of CD47 and band 3 in sucrose gradients are essentially identical in AA and CC erythrocyte samples, likely due to known interactions between these two proteins. This suggests that the more extensive band 3 clustering in CC erythrocytes may produce highly packed conditions for CD47, thereby reducing its detergent extractability as well.

### Change in repulsive force by zeta potential reduction in CC erythrocytes

We demonstrated that changes in the molecular profile and lateral organization of membrane molecules can alter charge distributions of the entire membrane. Treatment of normal AA erythrocytes with NaNO_2_ generated levels of membrane-bound hemichromes and ZP shifts that were comparable to those of CC erythrocytes, suggesting that hemichrome deposition may play a key role in remodeling CC erythrocyte membranes. The ZP difference between CC erythrocytes and NaNO_2_–treated AA erythrocytes is approximately 0.4 mV (2 mV *vs.* 1.6 mV, respectively). The larger ZP difference in CC erythrocytes may reflect the association of plasma proteins with CC erythrocyte membranes. These differences in ZP may also relate to the processes by which hemichromes are formed in these two cell types. In CC erythrocytes, hemichrome formation is a continuous process that occurs *in vivo* throughout the erythrocyte life-span. In contrast, hemichrome formation in chemically-oxidized AA erythrocytes *in vitro* occurs more rapidly. Also, the properties and membrane-altering effects of hemichromes may depend on whether they contain HbA or HbC.

We observed that negatively-charged PS was externalized at higher levels in a subset of CC erythrocytes. While these negative charges may not be sufficient to change the net charge of the erythrocyte surface, they may influence the membrane distributions of other charge-bearing molecules. Our results suggest that modified membrane microdomains contribute net positive charges that counteract increased negative charges derived from externalized PS on CC erythrocytes. In any case, decreases in ZP suggest that the repulsive forces between NaNO_2_-treated AA erythrocytes, CC erythrocytes, or other negatively charged cells are reduced. This may promote the interaction of CC erythrocytes with splenic macrophages that remove them from the circulation. This process may also occur with senescent AA erythrocytes as we observed reductions in ZP in older AA erythrocytes fractionated by ultracentrifugation. The bottom 25% fraction containing older erythrocytes displayed ≈1.2 mV more positive ZP than the top 25% fraction containing younger erythrocytes (not shown). Additional studies will be required to determine how the binding of hemichromes and plasma proteins to the inner membrane of CC erythrocytes contribute to the observed ZP alterations of these cells.
